# ST-elevation myocardial infarction complicated by ventricular tachycardia revealing coronary artery ectasia: a case report

**DOI:** 10.1186/s13256-023-03965-3

**Published:** 2023-06-06

**Authors:** Imane Tlohi, Fatiha Karim, Asmaa Elamraoui, Abdenasser Drighil, Rachida Habbal

**Affiliations:** Department of Cardiology, IBN ROCHD University Hospital, 1 rue des hopitaux 20360 Casablanca, Morocco

**Keywords:** Coronary artery ectasia, Acute coronary syndrome, STEMI, Coronary aneurysm, Ventricular tachycardia, Case report

## Abstract

**Background:**

Coronary artery ectasia is a rare angiographic finding and results from a disease process that compromises the integrity of the vessel wall. Its prevalence ranges between 0.3% and 5% of patients undergoing coronary angiography (Swaye et al. in Circulation 67:134–138, 1983). Coronary artery ectasia in patients with ST-elevation myocardial infarction is associated with an increased risk of cardiovascular events and death after percutaneous coronary intervention.

**Case presentation:**

We report the case of a 50-year-old male Caucasian patient, admitted for ventricular tachycardia at 200 beats per minute hemodynamically not tolerated that was reduced by external electric shock. Electrocardiogram after cardioversion showed a sinus rhythm with anterior ST-elevation myocardial infarction. Thrombolytic therapy was chosen after exposure to dual antiplatelet therapy and heparin since the expected time to percutaneous coronary intervention was greater than 120 minutes from first medical contact and the patient presented within 12 hours of onset of ischemic symptoms. The electrocardiogram after thrombolysis showed the resolution of the ST segment. The echocardiogram showed a dilated left ventricle with severe dysfunction with left ventricle ejection fraction at 30%. Coronary angiography revealed non-obstructive giant ecstatic coronaries without any thrombus. A check-up to look for possible etiologies for coronary artery ectasia was carried out and returned normal. Since no etiology for coronary artery ectasia was found at the limit of available exams in our center, the patient was discharged with antiplatelet therapy (aspirin 100 mg once a day) and heart failure treatment with an indication for an implantable cardiac defibrillator.

**Conclusions:**

Coronary artery ectasia in the context of acute myocardial infarction is a rare condition that may have dangerous complications, especially when an optimal treatment for ecstatic culprit vessels is still controversial.

## Background

Coronary artery ectasia (CAE) is defined as an arterial anomaly characterized by diffuse dilation, with a luminal diameter 1.5 times that of an adjacent normal segment, affecting more than 50% of the vessel length [1]. CAE is a rare entity, with a prevalence of approximately 0.3–4.9% in patients undergoing coronary angiography [2]. A distinction must be made between the term aneurysm, which refers to the local expansion of the coronary arteries, and the term ectasia, which is used to describe the elongation and dilation of tubular structures [3]. Atherosclerosis is the most common etiological mechanism of CAE. However, other possible causes have also been described in the literature, including congenital malformations, systemic inflammatory vasculitis, connective tissue diseases, genetic diseases, infectious diseases, and iatrogenic injury after percutaneous coronary intervention (PCI) [4]. Clinical manifestations range from incidental findings in asymptomatic patients, effort angina, exercise-induced ischemia to acute coronary syndrome [5]. Although the prognosis of CAE is still controversial, several studies have reported an increased risk of adverse events during long-term follow-up of patients with myocardial infarction and angiographic evidence of CAE [6]. Treatment of acute coronary syndromes in isolated CAE can be challenging because there is no consensus approach, and it still relies on physician selection on the basis of physicians’ own experience [7].

Here, we report the case of an ST-elevation myocardial infarction complicated by ventricular tachycardia revealing coronary artery ectasia in a male patient. We describe this rare condition because few similar cases have been reported in the literature, as well as to shed light on the management of this condition with fatal complications, especially in the absence of therapeutic consensus.

## Case presentation

A 50-year-old Caucasian man presented to our emergency room with chest pain lasting for 6 hours associated with palpitations and stage III New York Heart Association (NYHA) dyspnea. The patient’s cardiovascular risk factors included active smoking at 30 pack years. The patient had no past medical history, no coronary artery interventions, no history of illicit drug use or alcohol consumption, and he had never received any medication before this episode. There was no family history of ischemic heart disease or sudden cardiac death. The initial examination found a confused patient with a Glasgow Coma Scale at 14 without any sensory motor deficit. He was hemodynamically unstable, with blood pressure at 84/55 mmHg, heart rate of 200 beats per minute, respiratory frequency of 26 breaths per minute, and unremarkable cardiac auscultation.

**Fig. 1:**
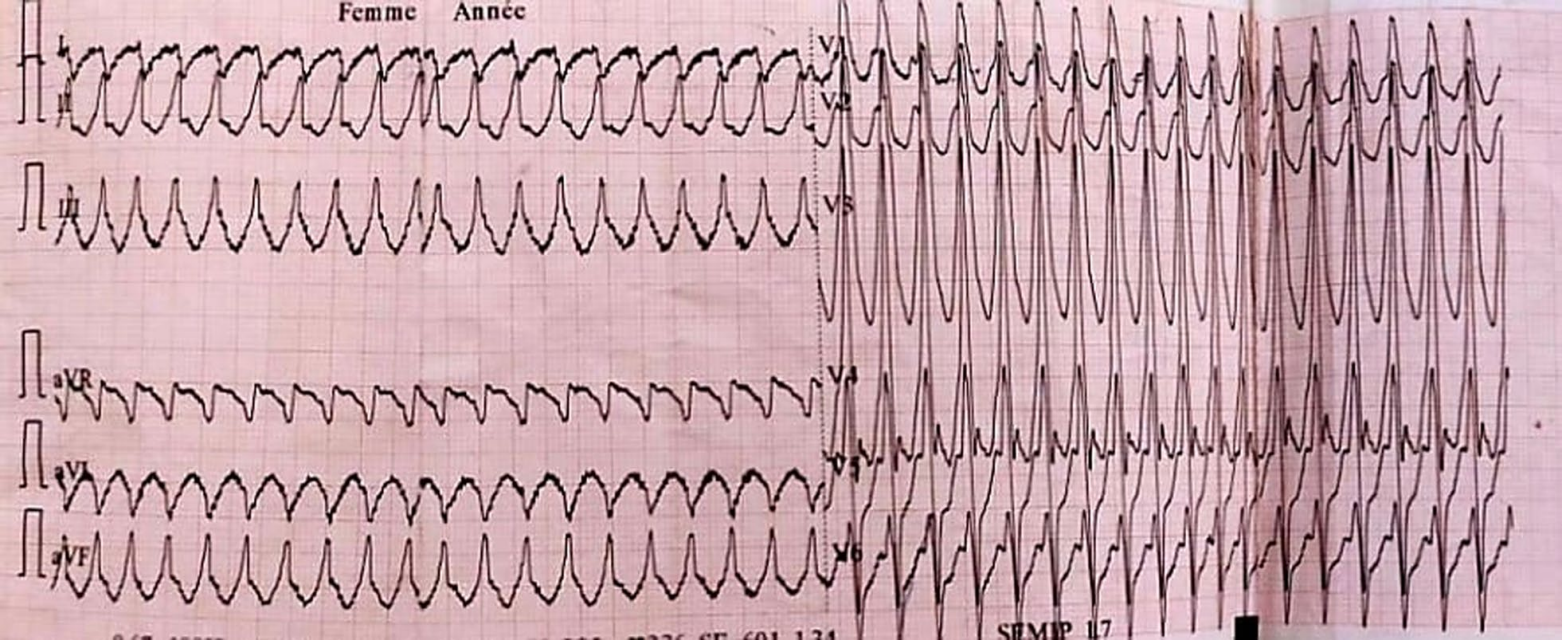
Initial electrocardiogram demonstrating ventricular tachycardia at 200 beats per minute

The initial electrocardiogram (ECG) showed ventricular tachycardia at 200 beats per minute reduced by an external electric shock at 200 J (Fig. 1).

**Fig. 2:**
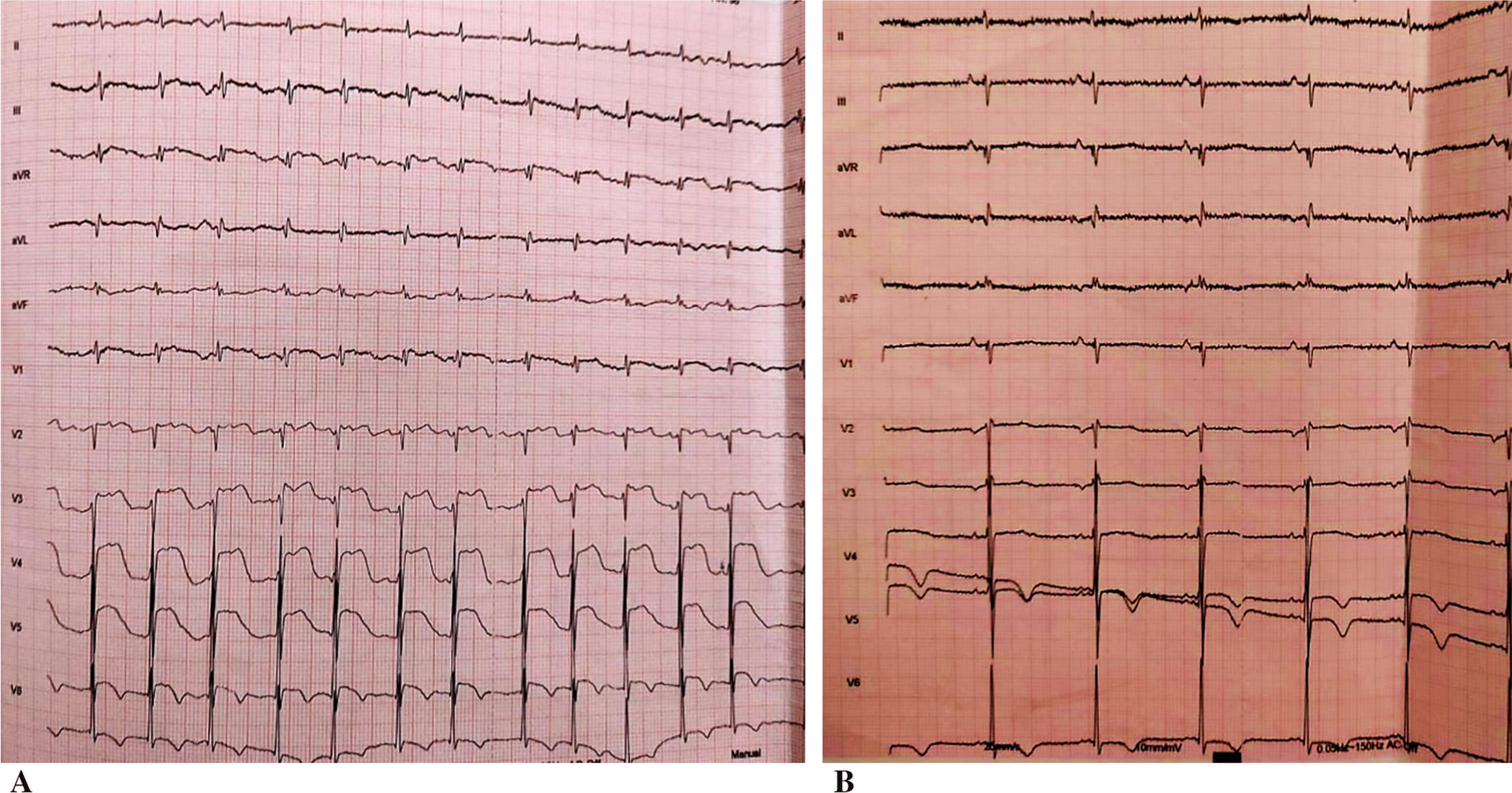
**A** Electrocardiogram after ventricular tachycardia reducing showing anterolateral ST-elevation myocardial infarction. **B** Post-thrombolysis electrocardiogram demonstration the resolution of ST segment elevation associated to negative T waves on apicolateral derivation

The post-reduction ECG showed a regular sinus rhythm with anterolateral ST elevation (Fig. 2A). Upon diagnosis of ST-elevation acute coronary syndrome, the patient was thrombolyzed with 50 mg of tenecteplase, as the patient was more than 120 minutes away from a percutaneous coronary intervention (PCI)-capable site. In addition to thrombolysis, the patient was also administrated intravenous heparin and oral dual antiplatelet therapy (DAPT) (aspirin 300 mg, clopidogrel 300 mg). The ECG carried out on post-thrombolysis observed the resolution of ST segment with negative T waves on the apicolateral derivations with a resolution of chest pain (Fig. 2B).

His laboratory tests showed a normal hemoglobin level at 15.8 g/dL, platelet count at 56,600, normal white blood cells (WBC) at 7.59 × 103/µL, and lymphocytes at 1.25 × 103/µL.

Biochemistry analysis revealed troponin I of 5.08 μg/L, C-reactive protein (CRP) at 72 mg/L, total protein of 69 g/L, albumin 35 g/L, alanine aminotransferase (ALT) 82 U/L, aspartate aminotransferase (AST) 168 U/L, triglycerides 0.66 mmol/L, and low-density lipoprotein (LDL) cholesterol 2.19 mmol/L. Serum electrolytes were normal.

Serum creatinine was normal and the estimated glomerular filtration rate [eGFR, calculated with the Modification of Diet in Renal Disease (MDRD) formula] was 89 mL/minute/1.73 m^2^. Urine culture was sterile.

The echocardiogram demonstrated a severely reduced left ventricular ejection fraction (LVEF) of 30% with severe global hypokinesis and a dilated left ventricle at 60 mm (indexed at 33 mm/m^2^).

**Fig. 3:**
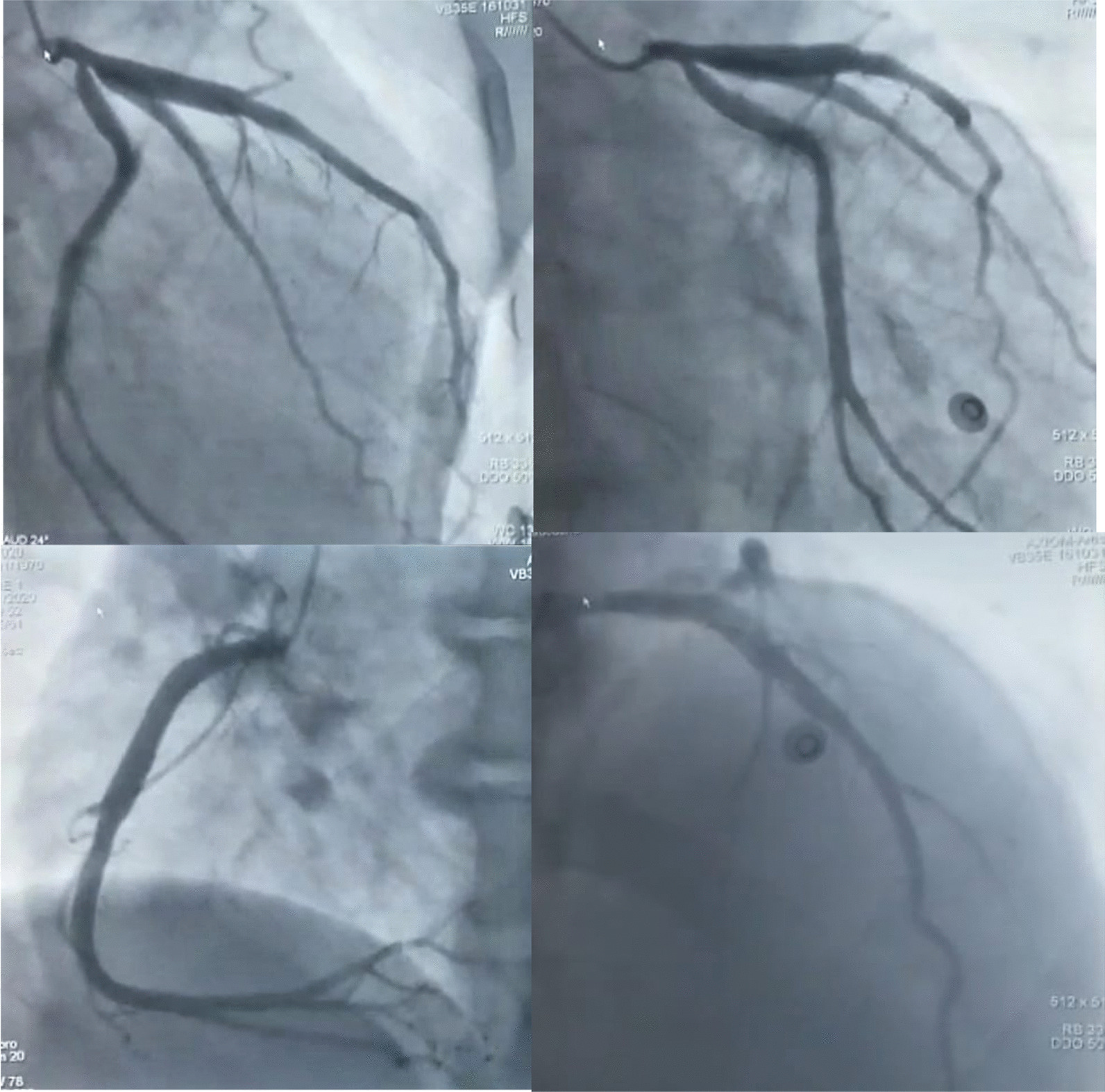
Coronary angiogram demonstrating ectatic coronary arteries

Coronary angiography demonstrated non-obstructive diffusely ectatic coronary arteries with no thrombus visualized and an insignificant lesion of the circumflex artery ostium (Fig. 3).

Tests in search of systemic inflammatory diseases and connective tissue disorders were carried out and came back negative.

A workup for viral serology [coronavirus disease 2019 (COVID-19), cytomegalovirus (CMV), Epstein Barr virus, coxsackie, herpes, Lyme disease, human immunodeficiency virus (HIV), and so on], for vasculitis, systemic disease, and bacterial infection (syphilis) was initiated and came back negative.

A magnetic resonance imaging (MRI) in this context was requested, showing a dilated cardiomyopathy with diffuse enhancement of the myocardium, evoking myocarditis.

During hospital stay and on discharge after 20 days, the patient was prescribed orally aspirin 100 mg daily with perindopril 2.5 mg daily, bisoprolol 2.5 mg daily, and spironolactone 12.5 mg daily for the management of heart failure with reduced ejection fraction with a view for uptitration to maximum tolerated dose. Faced with the alteration of the LVEF < 35% and the history of ventricular tachycardia, a single chamber implantable cardiac defibrillator was placed in our patient.

## Discussion

We shared the case of a 50-year-old male patient with no particular medical history, admitted for anterior ST-elevation myocardial infarction complicated by ventricular tachycardia at 200 beats per minute hemodynamically not tolerated that was reduced by external electric shock. Thrombolytic therapy was chosen and the electrocardiogram after thrombolysis showed the resolution of the ST segment. Coronary angiography revealed non-obstructive giant ecstatic coronaries without any thrombus and the echocardiogram showed a dilated left ventricle with severe dysfunction LVEF at 30%. A check-up to look for possible etiologies for CAE was carried out and came back normal. Since no etiology for CAE was found, at the limit of available exams in our center, the patient was discharged with antiplatelet therapy (aspirin 100 mg once a day) and heart failure treatment with an implantable cardiac defibrillator.

Coronary artery ectasia is a clinically asymptomatic condition and is often discovered accidentally on coronary angiography or computed tomography. Some reports indicate a higher prevalence in men [8]. Hypertension, dyslipidemia, smoking, and cocaine abuse have been associated with CAE [9, 10].

Markis *et al*., according to the extent of ectasia in the coronary tree, define a topographic classification of CAE: type I involving diffuse dilatation of two or three vessels (as described in our case report), type II represented by diffuse dilatation of one vessel and focal dilatation of another vessel, type III with diffuse dilatation of only one vessel, and type IV defined by a focal aneurysm [3].

The right coronary artery is the most frequently involved (40.4%), followed by left anterior descending (32.3%), left circumflex (23.4%), and less frequently left main coronary artery (3.5%) [11].

## Pathogenesis

The pathogenesis of CAD is not fully understood but may involve the destruction of the arterial media, thinning of the arterial wall, and increased wall stress [12]. Ectasia development is characterized by the overexpression of proinflammatory cytokines and enzymes that can degrade various structural proteins in the vessel wall. Extensive proteolysis of the extracellular matrix weakens the vascular structure, increases wall tension, reduces vascular resistance to blood flow, and predisposes progressive arterial dilatation [13-15]. Individual genetic susceptibility may be one of the contributing factors [8].

## Etiology

Atherosclerosis is the most common cause of CAE in adults, while Kawasaki disease is the most common etiology in childhood. Other systemic inflammatory diseases (such as Takayasu arteritis, systemic lupus, Behcet syndrome, and so on), infections (most commonly with staph aureus), connective tissue disorders (Marfan syndrome, Ehlers–Danlos syndrome, and so on), cocaine abuse, and traumatism after coronary interventions are other possible causes of CAE [8, 16].

## Clinical presentation and diagnosis

The clinical presentation of CAE is heterogeneous and varies from asymptomatic forms to acute coronary syndrome (ACS), effort angina, microvascular dysfunction, or compression of adjacent cardiac or noncardiac structures. Tamponade due to rupture of CAE is a rare but tragic complication.

Several mechanisms may explain the clinical event of ACS or effort angina in patients with CAE, including atherosclerotic plaque instability with high thrombus burden, distal embolization of thrombotic material, endoluminal thrombosis due to flow disturbances and blood stasis, in absence of underlying atherosclerotic lesions, or impairment of myocardial perfusion related to the severe slow flow. These two latter mechanisms are probably the most incriminated in the occurrence of acute coronary syndrome in our patient [8].

Coronary angiography represents the gold standard for diagnosing CAE. It detects the severe flow disturbances by local deposition of dye in the dilated coronary segment, segmental backflow, and delayed antegrade contrast filling [8]. Intravascular ultrasound (IVUS) may be used to examine higher vessel wall structure and consequently assist in differentiating between true aneurysms and pseudoaneurysms [18, 19].

Optical coherence tomography (OCT) has greater axial and spatial resolution compared with IVUS and can assess atherosclerotic plaque features, thrombus burden, and mechanisms of PCI failure in CAE [8, 20].

Noninvasive imaging tests can also diagnose CAE. Multidetector computed tomography and Magnetic resonance angiography provide important anatomical and functional information, especially in patients in whom other diagnostic procedures are contraindicated [21, 22].

Transthoracic and transesophageal echocardiography are useful in assessing the location of coronary artery anomaly (CAA) and the presence of intraluminal thrombi, especially in children with Kawasaki disease and large coronary aneurysms of the proximal left main artery and right coronary artery [8]. Its noninvasive nature makes it an ideal method for long-term follow-up of adult patients with a history of Kawasaki disease [8, 23].

## Management of CAE

Appropriate treatment of CAE remains controversial due to the lack of specific recommendations, limited case studies, anecdotal observations, and a lack of large randomized clinical trials. Potential treatment options include medical therapy, PCI, and surgery, but each of these strategies presents technical and clinical challenges [8].

Percutaneous treatment of CAE is a valuable option for patients with appropriate anatomy and clinical features. However, it is unclear whether conservative or interventional strategies are preferred in patients with CAE without obstructive coronary artery disease, and there is no evidence to support PCI results in this setting [8, 24]. Previous data showed that the presence of CAE was a risk factor for stent thrombosis after PCI in patients with acute coronary syndrome [25]. This can be explained by residual thrombus, turbulent flow, and stent malposition [8].

There is uncertainty about the indications for surgery in patients with CAE. However, surgical treatment should be considered as first-line therapy when PCI is considered high risk [8, 26]. Possible surgical procedures for coronary aneurysms include aneurysmectomy, ligation, or marsupial surgery with bypass surgery [8].

The optimal medical management of patients with CAE is still an area of ongoing debate. Modifying cardiovascular risk factors is mandatory since atherosclerosis is involved in the pathogenesis in most cases of CAE [8]. Though statins may be beneficial in inhibiting matrix metalloproteinases that cause arterial wall dilatation, this has not been demonstrated in prospective studies [27].

Aspirin administration has been recommended in the majority of patients with CAE and myocardial infarction on the basis of observation of concomitant obstructive coronary artery lesions [27]. Severe coronary dilatation is associated with impaired blood flow and blood stasis and predisposes to activation of the coagulation cascade with the risk of local thrombosis and distal embolization [8, 28]. However, the literature provides limited and conflicting evidence on this topic, especially in asymptomatic patients with incidentally discovered CAE [8].

The use of angiotensin-converting enzyme (ACE) inhibitors has been suggested for the treatment of CAE, as ACE gene polymorphisms have been found to be associated with the development of this disease, but their utility has yet to be proven [27].

Krüger *et al*. noted that the development of inducible ischemia in CAE depended on heart rate [17]. Therefore, β-blocker use has been proposed as a possible therapeutic option in patients because of their negative chronotropic effect and their reduction of myocardial oxygen demand in the absence of vasodilation [8]. However, their use has been questioned due to the risk of coronary spasms related to their unopposed α-receptors’ stimulation [27].

Calcium channel blockers may play a beneficial role in CAE by reducing the risk of coronary spasm and thrombus formation and improving coronary blood flow and myocardial perfusion [8, 29]. Trimetazidine also improves coronary blood flow and myocardial preconditioning by increasing adenosine levels, and thus may reduce exercise-induced myocardial ischemia in patients with CAE [30].

In patients with Kawasaki disease, the administration of intravenous immunoglobulin at the acute phase is associated with a high rate of regression of coronary aneurysms. OAC (oral anticoagulant) is recommended in selected patients with Kawasaki disease and large and/or rapidly expanding aneurysms [8, 31].

In the absence of a consensus for the treatment of CAE, patients should be regularly followed up to assess for new symptoms and treatment side effects.

## Conclusions

CAE is a rare condition and is usually diagnosed incidentally on coronary angiography. It is characterized by a variety of clinical manifestations ranging from asymptomatic cases to patients with high-risk ACS. Management of this entity is difficult in the absence of therapeutic consensus. The main treatment options include modification of CAD risk factors, anti-ischemic therapy, antithrombotic therapy, and percutaneous or surgical revascularization. Large multicenter trials and future studies are needed to optimize the management of this complex patient population.

## Data Availability

The datasets supporting this article are included in the article.

## Abbreviations

CAE: Coronary artery ectasia
ECG: Electrocardiogram
NYHA: New York Heart Association
DAPT: Dual antiplatelet therapy
MRI: Magnetic resonance imaging
PCI: Percutaneous coronary intervention
LVEF: Left ventricular ejection fraction
CAD: Coronary artery disease
CMV: Cytomegalovirus
HIV: Human immunodeficiency virus
IVUS: Intravascular ultra sound
OCT: Optical coherence tomography
ACS: Acute coronary syndrome
